# Small Molecular Peptides and Their Potential Antifungal Activities During the Pile-Fermentation of Post-Fermented Tea

**DOI:** 10.3390/foods15071263

**Published:** 2026-04-07

**Authors:** Xueli Pan, Mengyi Guo, Song Wu, Huan Huang, Yan Luo, Zhenjun Zhao, Xun Chen, Xianchun Hu, Huawei Wu, Xinghui Li

**Affiliations:** 1 College of Horticulture and Gardening, Yangtze University, Jingzhou 434025, China; 2 Fruit and Tea Research Institute, Hubei Academy of Agricultural Sciences, Wuhan 430072, China; 3 College of Life Science, Yangtze University, Jingzhou 434025, China; 4 College of Horticulture, Nanjing Agricultural University, Nanjing 210095, China

**Keywords:** small molecular peptide, post-fermented tea, pile-fermentation, antifungal potential

## Abstract

This study systematically investigated the dynamic diversity, potential sources, and antifungal activities of small molecular peptides during the pile-fermentation process of post-fermented tea. By analyzing the damaging effects of small molecular peptide extracts from tea samples at different pile-fermentation stages on the spore cell membranes of *Aspergillus carbonarius* (*A. carbonarius*) and the inhibitory activity against β-1,3-glucan synthase (β-1,3-GS), it was confirmed that some small molecular peptides exhibit significant antifungal effects. The main findings are as follows: (1) The number of identified small molecular peptides showed a trend of first increasing and then decreasing with the progress of pile-fermentation, peaking at 4453 species on the 35th day of pile-fermentation, and were dominated by hexapeptides and heptapeptides with molecular weights ranging from 600 to 800 Da. (2) Based on orthogonal partial least squares discriminant analysis (OPLS-DA), the samples were divided into three characteristic stages according to the differences in small molecular peptide composition at different stages, and 156 characteristic peptides with a relative abundance higher than 0.1% were screened out. Their precursor proteins were derived from 148 proteins belonging to 16 genera, including *Camellia*, *Aspergillus*, *Saccharomyces*, *Penicillium*, and *Bacillus*. (3) BLAST alignment results showed that five out of the 156 characteristic peptides were degradation fragments of known antifungal peptides originating from *Aspergillus* and *Bacillus*. (4) Combining molecular docking screening and in vitro verification of synthetic peptides, a total of 27 small molecular peptides with antifungal activity were obtained, and their mechanism of action was the inhibition of β-1,3-GS activity. (5) The small molecular peptides related to antifungal activity could be classified into two categories: enzymatic hydrolysates of known antifungal peptides, and the enzymatic hydrolysates of tea-derived proteins or macromolecular peptides. Both categories were mainly distributed in the three stages of pile-fermentation, and there was a significant positive correlation among the population size of dominant microorganisms, microbial peptidase activity, and the abundance of small molecular peptides. This study reveals the dynamic generation pattern and antifungal potential of small molecular peptides during the pile-fermentation of post-fermented tea, providing a new scientific basis for evaluating the dynamic changes in microbial communities in tea and effectively controlling the contamination of harmful fungi during the pile-fermentation process.

## 1. Introduction

Post-fermented tea (also known as dark tea), mainly produced in Yunnan, Hunan, Hubei, Sichuan and other regions of China [1], is regarded as one of the most distinctive and popular tea products. It is characterized by unique quality traits: brownish-red dry tea leaves, a thick, bright red infusion, a special aged aroma, and a sweet aftertaste [2]; as well as exerting effects on weight control, post-fermented tea also has the ability to inhibit fatty acid synthase and reduce total cholesterol levels [3,4]. The processing of post-fermented tea (different from that of black tea and green tea) was more complicated. Pile-fermentation, using sun-dried green tea as raw material, is the key process for the rapid formation of the unique color, aroma, taste and other quality attributes of post-fermented tea [5].

In the process of pile-fermentation, methods such as water spraying for humidification, covering for heat preservation, and pile turning for uniform mixing were employed to provide suitable temperature, humidity and nutrient conditions for facilitating the vigorous growth of various microorganisms [1]. The whole pile-fermentation process generally lasted for about 2 months or more, during which dominant microorganisms play a crucial role in mediating the enzymatic conversion of polyphenols, polysaccharides, proteins and other components in sun-dried green tea into polyphenol oxidation products, tea polysaccharides, tea pigments, and other flavor compounds of post-fermented tea through the secretion of extracellular enzymes [6,7]. For example, hydrolase (cellulase, protease, etc.) produced by dominant bacteria (e.g., *Bacillus* spp.), oxidases (polyphenol oxidase, peroxidase, etc.) secreted by dominant fungi (e.g., *Aspergillus* spp.) were found respectively at different pile-fermentation stages of post-fermented tea to promote the hydrolysis of polysaccharides and proteins, and the oxidation and polymerization of tea [8,9,10,11].

Along with the growth of dominant microorganisms represented by *A. niger* etc., during the pile-fermentation of post-fermented tea, there were also lots of other kinds of microorganisms, especially some harmful fungi, which grew and reproduced in tea through natural inoculation or some hitherto unknown forms of transmission [12,13]. It is generally accepted that the growth and proliferation of harmful fungi during pile-fermentation pose potential risks to the safety of post-fermentation tea consumption [14]. *Aspergillus versicolor*, *A. penicillioides* and *A. ochraceus* in Pu-erh tea [15,16], *A. glaucus* and *A. ochraceus* in Liubao tea [17], some strains belonging to *Eurotium* in Fuzhuan tea [18], *A. carbonarius* in Kangzhuan tea [19], *A. fumigatus* in Qingzhuan brick tea [20] and other harmful fungi were often detected and had the potential to produce mycotoxins such as aflatoxin, ochratoxin and fumagillin [21], and the detection of these harmful fungi during the pile-fermentation of post-fermented tea has attracted widespread attention. However, there is little concern regarding the safety risks of post-fermented tea consumption associated with harmful fungi, as numerous studies have demonstrated that various bioactive components in tea can effectively inhibit the growth of harmful fungi and the production of related mycotoxins. For example, gallic acid could inhibit the growth of *A. carbonarius* [22], tea polyphenols and catechin monomers (such as quercetin) can have a good inhibitory effect against the growth of *A. flavus* and can inhibit the production of aflatoxin by regulating the expression of the *aflR* gene related to aflatoxin synthesis [23,24,25].

In our previous studies, different species of *Bacillus* (*B. subtilis*, *B. licheniformis*, *B. laterosporus*, *Bacillus coagulans*, *B. pumilus*, *Brevibacillus brevis*) derived from the pile-fermentation process of post-fermented tea had been found to have significant inhibitory effects on the growth and mycotoxin production of ochratoxin-producing fungi during their symbiosis with ochratoxigenic fungi [2]; subsequent studies further revealed that it was the peptides produced by *B.* spp. that play a significant inhibitory role on the fungal growth and mycotoxin production [26]. Berglund et al. [27] also reported that peptides produced by some fungi can inhibit fungal growth. The discovery of a 58-amino-acids antifungal peptide produced by *A. niger* further provided strong evidence that fungi can produce antifungal peptides [28]. In addition, enzymatic hydrolysis of various natural proteins (e.g., gliadin and litsea cubeba protein) is also considered an important way to obtain antifungal peptides [29,30]. All of the above reports reminded us that while the often-mentioned active components of tea (e.g., tea polyphenols, etc.) might have antimicrobial effects, the peptides secreted by microorganisms (such as *Bacillus* etc.) or peptides formed by the degradation of tea proteins via microbial enzymatic hydrolysis during the pile-fermentation of post-fermented tea also merit greater attention, and the role of these peptides in mitigating safety risks from harmful fungi during this process warrants further in-depth investigation. Since protease or peptidase produced by microorganisms often lead to the further hydrolysis of peptides to form small molecular peptides during the pile-fermentation process, small molecular peptides may be an important witness of peptides secreted by microorganisms or peptides formed by degradation of proteins during the pile-fermentation process. Therefore, it is necessary to study the diversity of small molecular peptides to reveal the composition of peptides at different pile-fermentation stages of post-fermented tea and their potential antifungal activities, which will provide new ideas for people to reveal the mechanism of the low safety risk of drinking post-fermented tea caused by harmful fungi from the perspective of dynamic production of peptides and dynamic inhibitory effects of peptides against harmful fungi in the pile-fermentation process.

The purpose of this study was to reveal the dynamic changes in small molecular peptides and to identify the peptides with antifungal potential in the pile-fermentation process of post-fermented tea, which not only contributed to further enriching the research of active substances with antimicrobial potential in tea, but also expanded a new idea for biological control of harmful fungi in the pile-fermentation process of post-fermented tea. In this study, Qingzhuan brick tea was taken as the representative of post-fermented tea. The dynamic changes in small molecular peptide compositions during pile-fermentation (sampled at 0, 7, 14, 21, 28, 35, 42, 49 d) were systematically investigated by the label-free quantification technology with nanoLC MS/MS, and the potential biological sources of small molecular peptides in tea samples at different pile-fermentation stages were revealed using peptidomics combined with microbiome data. In addition, based on the analysis of the antifungal activity of small-molecule peptides extracts during pile-fermentation of post-fermented tea, small molecular peptides with potential antifungal activity were identified by comparison with known antimicrobial peptides from the antimicrobial peptide website, or by molecular docking (further validation by enzymatic methods based on inhibition activity of synthetic peptides on β-1,3-GS).

## 2. Materials and Methods

### 2.1. Materials

**Figure 1:**
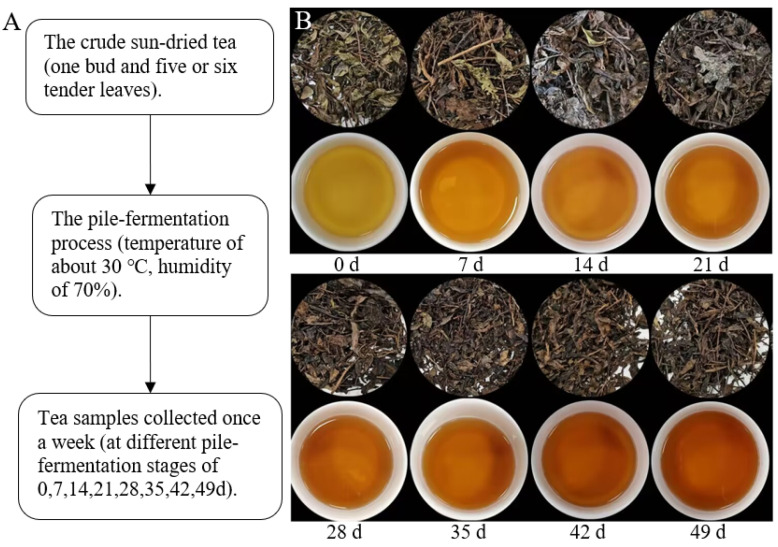
Characteristics of tea samples gathered during pile-fermentation process. (**A**) Requirement of pile-fermentation process and sampling plan; (**B**) dried tea and tea soup of tea samples from different pile-fermentation stages.

Crude sun-dried tea was prepared from one bud and five to six tender leaves of Camellia sinensis cv. Fuding was used as the raw material for post-fermented tea pile-fermentation. The fermentation process was conducted at Hubei Dongzhuang Tea Co., Ltd. (Chibi, China) following the schematic diagram presented in Figure 1. Briefly, the crude sun-dried tea was sprayed with water to adjust the moisture content to approximately 40% and then incubated in a controlled environment favorable for microbial growth (temperature: ~30 °C, humidity: ~70%) for approximately 7 weeks. Tea samples in the middle layer of the tea pile were collected once a week for subsequent analysis. A total of 8 sample groups were collected, corresponding to pile-fermentation time points of 0, 7, 14, 21, 28, 35, 42, and 49 days respectively. Triplicate samples were collected at each time point.

*A. carbonarius* H9, from a previous study [2], was an OTA-producing fungus isolated from the pile-fermentation process of post-fermented tea and was used as an indicator fungus for subsequent experiments of inhibition activity of peptide extracts on fungi.

Deionized water was generated using a Milli-Q system (Millipore, Bedford, MA, USA). MS-grade solvents for LC-MS analysis, including acetonitrile and methanol, were purchased from Tianjin Siyou (Tianjin, China). Pure standard reagents were purchased from Sigma (St. Louis, MO, USA). The synthetic peptides used in this study were synthesized by Shanghai Qiangyao Biotechnology Co., Ltd. (Shanghai, China). Unless stated, all other chemical reagents were analytical grade and provided by Sinopharm Group (Beijing, China).

### 2.2. Extraction of Peptides in Tea Samples from Different Pile-Fermentation Stages

Peptide extraction from tea samples was performed as described by Mathias Salger et al. and Zhang et al. with minor modifications (detailed below) [31,32]. First, tea samples (supplemented with 10% PVPP based on tea weight) were rapidly ground into a fine powder in liquid nitrogen, and the appropriate amount of tea sample powder was weighed into a 50 mL centrifuge tube. Following three extractions with n-butane to remove lipid-soluble components, three volumes of Tris-HCl buffer (50 mM, pH 8.0) containing a lysis solution (2.5 g SDS/100 mL, 7 mol·L^−1^ urea, and 2 mol·L^−1^ thiourea) was added. The mixture was oscillated in an ice bath for 2 h for extraction. Then, the supernatant was obtained by centrifuging the mixture at 12,000 RPM and 4 °C for 30 min. Four volumes of pre-cooled (−20 °C) solvents (acetone, dichloromethane, methyl acetate, ethyl acetate, and n-butyl acetate) were sequentially added to the supernatant. The mixture was incubated at −20 °C to remove caffeine, catechins, tea polyphenols, and pigments. Thereafter, the precipitate was redissolved in Tris-HCl buffer (50 mM, pH 8.0) (excluding lysate), and the supernatant was obtained by centrifugation (8000 rpm, 5700× *g*, 15 min). Finally, the supernatant was purified by desalt column and polyethersulfone ultrafiltration membrane filter (1 kDa, Sartorius, Shanghai, China) to obtain peptide extracts with molecular weight around 1 kDa. The peptide extracts were freeze-dried and stored at −20 °C for subsequent peptidomics analysis. Bradford G-250 reagent [33] was used to estimate the content of small molecular peptides.

### 2.3. Peptidomics Analysis of Small Molecular Peptides

#### 2.3.1. nanoLC MS/MS Analysis of Small Molecular Peptides

The extract of 2 µg small molecular peptides was redissolved in deionized water (with 0.1% formic acid, 2% acetonitrile) containing synthetic peptide (Val-Gln-Leu-Ala-Gly-Pro) as the internal standard and was separated and analyzed with a nano UPLC (EASY nLC1200) coupled to a Orbitrap Fusion Orbitrap instrument (Thermo Fisher Scientific, Waltham, Massachusetts, USA) with a nanoelectrospray ion source as described by Zhu et al. [34]. A reversed-phase C_18_ column (0.1 × 150 mm id, 1.9 μm particle size, Reprosil Pur 120 C18 AQ, Dr. Maisch, Ammerbuch-Entringen, Germany) was used for peptide separation. Mobile phases were H_2_O with 0.1% FA (formic acid), 2% ACN (acetonitrile) (phase A) and 80% ACN, 0.1% FA (phase B). Samples were separated at a flow rate of 300 nL/min within 120 min using the following gradient mode. Gradient mode: 4–35% for 105 min, 35–70% for 8 min, 70–100% for 1 min, and 100% for 6 min.

Data were collected with Data-dependent acquisition (DDA) mode in positive ion mode by Orbitrap analyzer (at a resolution of 120,000) (@200 m/z). Full scan (MS1) range: 400–1600 m/z for secondary scan (MS2); the resolution was set to 15,000 with a fixed first mass of 110 m/z. The automatic gain control (AGC) target for MS1 was set to 1 × 10^6^ with maximum ions implantation time (max IT) 30 milliseconds (ms), and 1 × 10^5^ for MS2 with max IT 100 ms. The top 20 most intense ions were fragmented by HCD with normalized collision energy (NCE) of 35%, and isolation window of 1.6 m/z. According to the chromatographic peak width, the dynamic exclusion time was set to 45 s; ions with single charge and >6 valence were excluded from the DDA procedure.

#### 2.3.2. Identification and Homology Analysis of Small Molecular Peptides

The nanoLC MS/MS data were processed using Proteome Discoverer (PD) software (Version 2.4.0.305, Thermo Fisher Scientific) and the built-in Sequest HT search engine for peptide identification and quantification. The false discovery rate (FDR) was set to 0.01 for both Peptide–Spectrum Matches (PSM) and peptide levels. Peptide identification was performed with an initial precursor mass deviation of up to 10 ppm and a fragment mass deviation of 0.02 Da. Unique peptide and Razor peptide were used for peptide quantification and total peptide amount for normalization. All the other parameters were reserved as default values. The identified peptide sequences were locally searched and compared in the Swiss Prot database or the genera level UniProt FASTA databases (uniprot-*Camellia sinensis*-4442-2023-03-06.fasta; uniprot-*Bacillus*-sub.fasta; uniprot-*Penicillium ucsense*-1181-2023-07-24.fasta; uniprot-*Aspergillus* sp.-2442-2023-07-24.fasta; uniprot-*Saccharomyces cerevisiae*-2311-2023-07-24.fasta and other available proteome databases of microorganisms) to find their homologous sequences for the further biological origin analysis of small molecular peptides.

### 2.4. Inhibitory Activity of Peptide Extracts on Fungi

In this study, the strain of *A. carbonarius* H9 was used as an indicator fungus to study the antifungal activity of peptide extracts from the following two aspects:

***Inhibitory activity of peptide extracts on* β-1,3-GS *of A. carbonarius***

The extraction of β-1,3-Glucan synthase from *A. carbonarius* was referred to the method reported by Zhang et al. [35]. Briefly, *A. carbonarius* H9 was cultured in PDB medium (25 °C) for 5 days and then mycelia was obtained by vacuum filtration. After freeze-drying, an appropriate amount of mycelium was immersed in a given volume of sodium acetate buffer (50 mmol/L, pH 5), crushed using an Ultrasonic Cell Disruption System (400 W, 10 min) in an ice bath, and then centrifuged at 8000 rpm (5700× *g*), 4 °C for 15 min to obtain the supernatant as the crude extract of β-1,3-GS. Then, 1.0 mL of the crude enzyme solution was mixed with 2 mL of peptide extracts. After the mixture was left for 10 min, the activity of GS was measured. The enzyme activity was expressed in enzyme/mg. The specific methods for the determination of enzyme activity were carried as reported by Hou et al. [36].

***Effect of peptide extracts on membrane permeability of fungal spore***

Effects of peptide extracts on the spore membrane integrity of *A. carbonarius* H9 was studied according to Zhao et al. [26] with some modifications. A total of 90 μL 1 × 10^7^ CFU/mL spore suspension of *A. carbonarius* H9 (diluted with 5% PDB medium) and 10 μL peptide extracts were loaded into 1.5 mL centrifuge tube and sterile water was added until the final concentration of the peptide extract in 1.5 mL centrifuge tubes was 0.05 mg/L. In the control group, sterile water was added instead of peptide extracts. After 6 h of incubation at 25 °C, 500 mg/L propidium iodide (PI) storage solution was added to reach a final concentration of PI at 50 mg/L. Fungal spores were observed under fluorescence microscope and photographed: the excitation wavelength and emission wavelength of PI were respectively 535 nm and 615 nm. Each treatment was repeated three times. Three fields were randomly selected for each slide, and photos were taken under fluorescent field and bright field at the same position. The number of spores observed in the bright field was defined as the total number (*T*), and the number of spores observed in the fluorescence field that were stained red (spores with damaged membrane structure) was denoted as (*S*). The inhibition rate of small molecular peptides on fungal spores, that is, the damage degree to spore membrane of *A. carbonarius* by small molecular peptides was expressed by cell membrane permeability rate of spore, which was calculated according to Formula (1).

(1) Membrane permeability rate of spore=ST×100

### 2.5. Identification and Quantitative Analysis of Antifungal Peptides in Major Peptides

The peptide sequences were entered into antimicrobial peptide database (APD, https://aps.unmc.edu/) and proteome database for the homologous search and alignment to screen and identify antimicrobial peptides or antimicrobial peptide degradation fragments. Quantitative analysis of antifungal peptides or antifungal peptide fragments was performed by calculating the relative abundance between antifungal peptides or antifungal peptide fragments and synthetic peptides based on mass spectrometry data using synthetic peptide (Val-Gln-Leu-Ala-Gly-Pro) as internal standard.

### 2.6. Analysis of Potential Antifungal Activities of Other Major Small Molecular Peptides

#### 2.6.1. Screening of Small Molecular Peptides with Potential Antifungal Activity Through Molecular Docking with β-1,3-Glucan Synthase

According to the results of mass spectrometry identification and inhibitory activity of peptide extracts, small molecular peptides with high abundance were used for docking with β-1,3-GS to initially screen the peptides with potential antifungal activity. The specific operation method was referred to as described by Zhang et al. [35].

#### 2.6.2. Verification of Antifungal Potential of Small Molecular Peptides by Inhibiting β-1,3-GS with Synthetic Peptides

To ensure the consistency and comparability of antifungal activity verification, all synthetic peptides were tested at a uniform mass concentration of 0.05 mg/mL. It should be noted that due to the differences in molecular weights of the peptides (ranging from 579 to 830 Da), their corresponding molar concentrations vary. The molar concentration of each peptide was calculated using the Formula (2).

(2) $$Molar\;Concentration\left(\mu M\right)=\frac{Mass\;Concentration\left(\frac{mg}{mL}\right)\times 10^{6}}{Molecular\;Weight(Da)}$$

Small molecular peptides with binding energy ranged from −0.54 to −9.01 kcal/mol to GTPase identified by molecular splicing were synthesized for further verification of their antifungal activity. The antifungal activity of small molecular peptides was expressed by the inhibition activity of small molecular peptides on β-1,3-GS of *A. carbonarius.* For specific methods, see Section 2.4.

### 2.7. Statistical Analysis

Statistical analyses were conducted using SPSS statistics software (version 26, IBM Corp., Armonk, NY, USA), and the data were tested using one-way analysis of variance (ANOVA) and Duncan tests; the differences were considered significant at *p* < 0.05. Data charts were prepared using R language (version 4.4.1), Simca (version 14.1), GraphPad Prism (version 9.5.1), Origin (version 2024, OriginLab, Northampton, MA, USA) and TBtools-II (version 1.120) statistical software.

## 3. Results and Discussion

### 3.1. Content and Antifungal Activity of Small Molecular Peptides in Tea Samples During the Pile-Fermentation

#### 3.1.1. Changes in the Content of Small Molecular Peptides During the Pile-Fermentation

**Figure 2:**
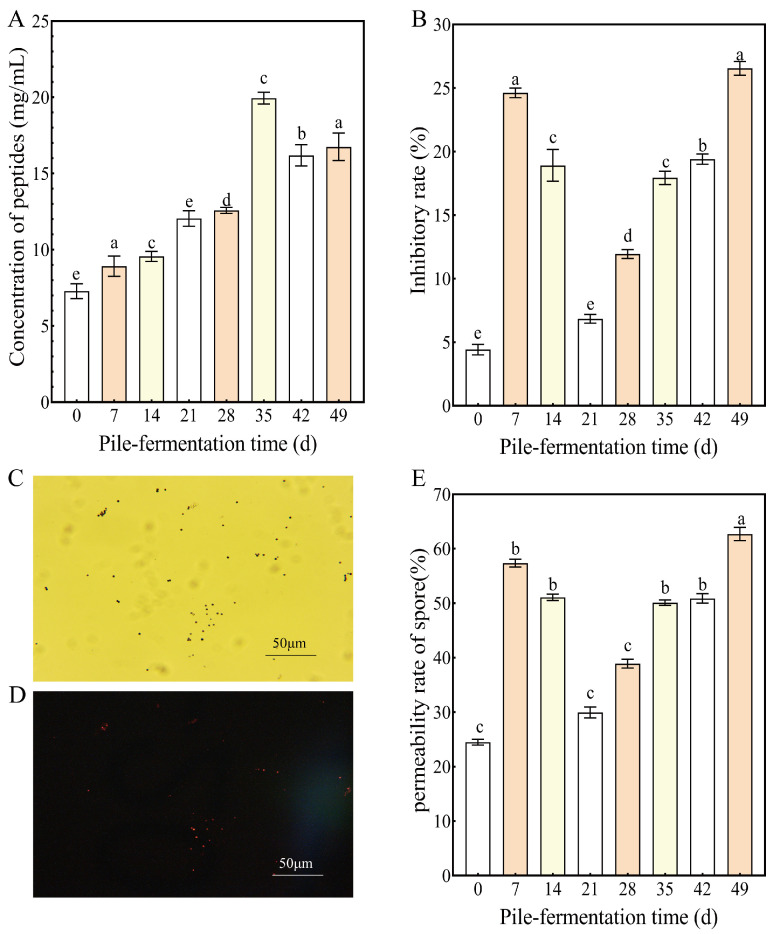
The content and antifungal activity of small molecular peptides in tea samples at different pile-fermentation stages. (**A**) The content of peptides; (**B**) inhibition rate of peptide extracts on β-1,3-glucan synthase; (**C**,**D**), fluorescence micrograph of spores treated by 0.05 mg/L peptide extracts (on 7 d of pile-fermentation) under bright field and fluorescent field, respectively; (**E**) effect of peptide extracts on membrane permeability of *A. carbonarius* spores. The letters a, b, c, d, and e on the bar chart are all significance markers for differences; different letters indicate significant differences between groups (*p* < 0.05), while the same letters indicate no significant differences between groups (*p* > 0.05).

Peptides, widely present in various organisms, are a common class of organic compounds formed by linking multiple amino acids via peptide bonds. They not only play a vital role in biological processes [37], but also are regarded as key characteristic components contributing to the flavor quality of tea [38]. In this study, peptide extracts of tea samples from eight different pile-fermentation stages of post-fermented tea were ultra-filtrated using a 1 kDa ultrafiltrate membrane to obtain small molecular peptides with molecular weight of around 1 kDa for the subsequent peptidomic and antifungal activity analysis. The mass concentration of small molecular peptides in tea samples gradually increased with the progression of pile-fermentation, ranging from 7.28 ± 0.48 mg/mL to 19.94 ± 0.39 mg/mL. The concentration of small molecular peptides at 35 d of pile-fermentation reached its maximum and then decreased significantly at 42 d and 49 d of pile-fermentation, as shown in Figure 2A. The initial increase in peptide mass concentration during pile-fermentation was attributed to the degradation of tea proteins, whereas the subsequent decrease reflected extensive peptide degradation in the later stages of fermentation. The instability of peptides was similar to that of tea polysaccharides, tea polyphenols, amino acids and other functional components in tea during the pile-fermentation, which all showed the transformation or degradation process under the action of microbial enzymes [39].

#### 3.1.2. Antifungal Activities of Small Molecular Peptides Extracted from Tea Samples at Different Pile-Fermentation Stages

β-1,3-glucan is recognized as a key component in constructing the core skeleton of the fungal cell wall, and β-1,3-GS plays a critical role in the catalytic synthesis of β-1,3-GS [40]. β-1,3-GS was often used to evaluate the inhibitory effect of peptides against fungal growth. The stronger the inhibitory activity of peptides against β-1,3-GS, the more potent their inhibitory effect on fungal growth [35]. In this study, β-1,3-GS extracted from the mycelium of *A. carbonarius* was used to evaluate the antifungal activity of peptide extracts from tea samples at different pile-fermentation stages; the inhibitory effect of peptide extract on β-1,3-GS activity is shown in Figure 2B. The results showed that the peptide extracts (0.05 mg/mL) from the tea samples at different pile-fermentation stages exhibited an inhibitory effect (inhibition rate ranged from 4.42% to 25.38%) on the β-1,3-GS, which indicated that the peptide extracts could inhibit the catalytic synthesis of β-1,3-glucan (an essential ingredient of the fungal cell wall) and thus to realize the inhibition of fungal growth. The inhibitory activity of peptide extracts against β-1,3-GS increased with the prolongation of pile-fermentation time and exhibited stage-specific differences. In particular, the inhibitory activities of peptide extracts in tea samples on 7–14 d and 35–49 d of pile-fermentation were significantly enhanced compared with that of peptide extracts at other pile-fermentation stages, which could be related to the dynamic changes in microbial diversity during pile-fermentation. Based on previous data on microbial community dynamics during pile-fermentation [9], the increase in peptide inhibitory activity coincided with the rapid growth and proliferation phases of microorganisms such as *Bacillus* sp. and *Aspergillus* sp. Specifically, a larger population of dominant microorganisms (e.g., *Bacillus* sp.) was associated with higher inhibitory activity of peptide extracts against β-1,3-GS during fermentation. This seems to prove once again that microorganisms such as *Bacillus* can produce antimicrobial peptides and play an important role in the inhibition of fungi [2,35]. Furthermore, theaflavins, thearubigins and other substances formed via the oxidation of tea catechins during fermentation, as well as organic acids (e.g., lactic acid, acetic acid) generated during fermentation, have been confirmed to disrupt fungal cell membrane potential [41,42]. Such membrane damage can increase the permeability of antifungal peptides into fungal cells, thereby enhancing the antimicrobial activity of these peptides.

The inhibition of peptides on fungal growth was also reflected in the destruction of the fungal spore cell membrane by peptides [2]. Propidium iodide (PI) can penetrate damaged cells and bind to nucleic acids, emitting red fluorescence when the fungal spore membrane is compromised [43]. The percentage of PI-stained fungal spores represents the fungal spore membrane disruption rate, which serves as a visual method for assessing the inhibitory effect of peptides against fungal growth [44,45]. The study showed that the integrity of the cell membrane of *A. carbonarius* H9 spores treated with peptide extracts (with a concentration of 0.05 mg/mL) from the tea samples at different pile-fermentation stages was significantly reduced, indicating that the peptide extract showed significant damage to the cell membrane of *A. carbonarius* H9 spores, and the damage rate of the cell membrane ranged from 24.58% to 62.70%. Figure 2C,D shows the fluorescence staining effect of *A. carbonarius* spores treated with peptide extracts on the 7th day of pile-fermentation, with a staining rate of 57.32%. The damage effect of the peptide extracts at different pile-fermentation stages on the cell membrane of *A. carbonarius* spores was similar to the inhibitory effect of the peptide extracts on β-1,3-GS (in Figure 2E). The damage effect of peptide extract on the spore membrane of *A. carbonarius* was first enhanced (the fluorescence staining rate of spores treated with peptide extracts on 7th day of pile-fermentation reached a higher 57.32%), then decreased (from 14 d to 21 d of pile-fermentation), and finally increased (from 14 d to 21 d of pile-fermentation, the fluorescence staining rate of spore treated with peptide extract after 49th day of pile-fermentation was 62.70%). Neither the damage effect of peptide extracts on the cell membrane of *A. carbonarius* spores nor the inhibition effect on β-1,3-glucan synthetase were consistent with the trend of the gradual increase in small-molecule peptides content in the process of pile-fermentation, indicating that only a part of the peptides with antibacterial effect in the total peptide extracts played a role in the damage effect on the cell membrane of *A. carbonarius* spores and the inhibition effect on β-1,3-GS. In this study, the results of the fluctuation changes in the damage effect of peptide extracts on the cell membrane of *A. carbonarius* spores and the inhibition effect of peptide extracts on β-1,3-GS synthetase at different stages of pile-fermentation further illustrate the dynamic changes in peptides with antimicrobial activity during the pile-fermentation of post-fermented tea: both the increase in antimicrobial peptides content was caused by the growth and reproduction of specific microorganisms during the pile-fermentation process, and the degradation of antimicrobial peptides was caused by peptidases produced by some microorganisms in the pile-fermentation process.

### 3.2. Dynamic Changes in Small Molecular Peptides in Tea Samples at Different Pile-Fermentation Stages of Post-Fermented Tea

#### 3.2.1. Number of Small Molecular Peptides Identified in Tea Samples from Different Pile-Fermentation Stages

**Figure 3:**
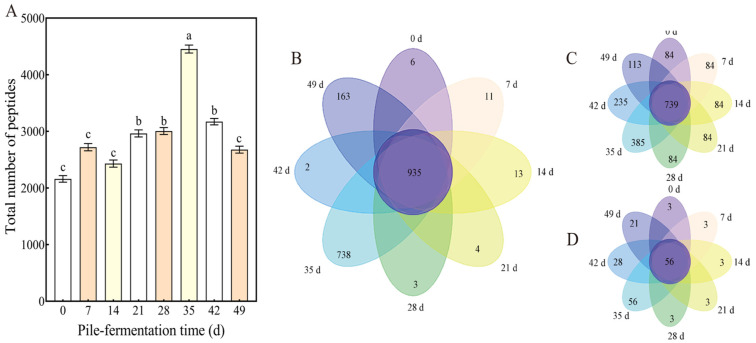
Changes in peptide number in tea samples at different pile-fermentation stages. (**A**) The total number of peptides; (**B**) Venn diagrams of the total peptide number in tea samples of 0, 7, 14, 21, 28, 35, 42 and 49 d; (**C**) Venn diagrams of peptide number (with amino acid numbers between 6 and 9) in tea samples of 0, 7, 14, 21, 28, 35, 42 and 49 d; (**D**) Venn diagrams of peptide number (with amino acid numbers ≥ 10) in tea samples of 0, 7, 14, 21, 28, 35, 42 and 49 d. The letters a, b and c on the bar chart are all significance markers for differences; different letters indicate significant differences between groups (*p* < 0.05), while the same letters in-dicate no significant differences between groups (*p* > 0.05).

The compositions and dynamic changes in peptides in tea samples from different pile-fermentation stages of post-fermented tea were identified and analyzed by LC-MS/MS, mass spectrometry software (Proteome Discoverer 2.4) and an available online peptide database [32]. Analysis of the peptide count in fermented tea samples at different pile-fermentation stages was conducted, and the results are shown in Figure 3A. The study revealed that a total of 2160 peptides were detected at the initial stage of pile-fermentation (0 d), which gradually increased to a peak of 4453 at 35 d. The number of identified peptides in tea samples then began to decrease during the late fermentation stage (42–49 d). Previous studies have identified 39 short peptides and 83 long peptides in black tea [46]; 228 and 809 peptide fragments in fermented dairy products (goat milk kefir and cheese, respectively) [47]; and He et al. detected 674 peptide-containing substances in commercial soy sauce, including 215 dipeptides [48]. In contrast, the number of peptide fragments in this study (up to 4453) was markedly higher than those in black tea, fermented dairy products (goat milk kefir, cheese) and soy sauce. This further demonstrates the uniqueness of the post-fermented tea pile-fermentation process. The trend in the number of identified peptides during the entire pile-fermentation was consistent with the trend of peptide content and showed a similar staged variation to other major tea quality components such as tea polyphenols, under the action of endogenous enzymes and moist heat [39]. The differences in peptide sequences in tea samples from different stages of pile-fermentation (at 0, 7, 14, 21, 28, 35, 42 and 49 d) were analyzed by means of Venn diagram (Figure 3B–D). The results showed that, although the content and quantity of peptides in tea samples from different pile-fermentation stages varied greatly due to the influence of various pile-fermentation conditions and other factors, the sequence of many peptides identified in tea samples from different pile-fermentation stages remained the same. For example, 935 peptides with the same sequence could be identified in tea samples from different pile-fermentation stages, of which 739 were peptides containing 6–9 amino acids and only 56 peptides contained more than 10 amino acids. The peptides with the same sequence in tea samples from different pile-fermentation stages were found to derive from endogenous peptides or were produced by degradation of the same precursor protein [49]. Compared with the peptides identified in tea samples at other pile-fermentation stages, the number of specific peptides (with different sequences) in tea samples at pile-fermentation of 35 d was the largest, with 738 peptides that were not identified in tea samples at other pile-fermentation stages, among which there were 385 peptides with 6–9 amino acids and 56 peptides containing more than 10 amino acids (Figure 3C,D). Previous studies reported that peptide diversity in white tea varies with natural withering, which is attributed to the degradation of tea proteins by endogenous hydrolases during processing [50]. Additionally, peptide diversity is widely considered to result from the enzymatic degradation of tea proteins by microorganisms [32]. Different microorganisms secrete different hydrolases in the process of their growth and metabolism, and different peptides will be produced in the same plant substrate under the action of different hydrolases [51,52]; thus, it can be thought that the formation of peptide diversity in tea samples at different pile-fermentation stages may be a prominent reflection of the differences in different microbial populations and their dynamic metabolic processes during the pile-fermentation process.

#### 3.2.2. Molecular Weight of Small Molecular Peptides in Tea Samples from Different Pile-Fermentation Stages

**Figure 4:**
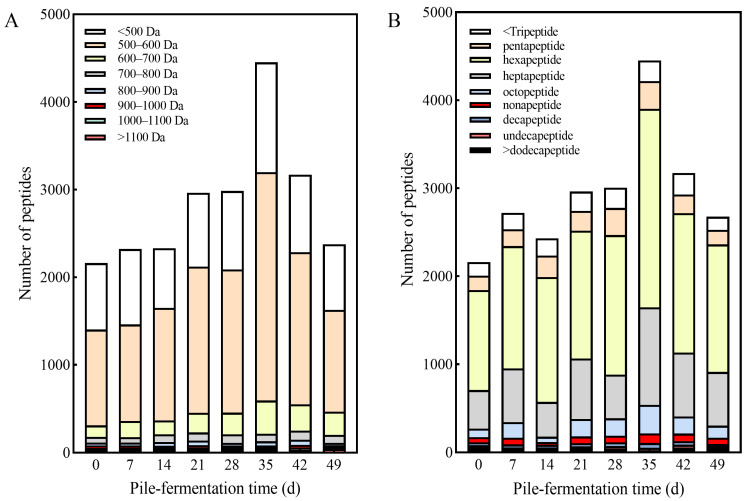
Molecular weight and chain length distribution of the peptides identified in tea samples at different pile-fermentation stages of post-fermented tea. (**A**) Peptide molecular weight distribution; (**B**) peptide chain length distribution.

The molecular weight of the peptides identified in tea samples from different pile-fermentation stages was mainly distributed in three ranges of 600–700, 500–600 and <500 Da; the number of peptides with a molecular weight of 500–600 Da > peptides with a molecular weight less than 500 Da > peptides with a molecular weight of 600–700 Da (Figure 4A). Among them, the number of peptides with a molecular weight of 500–600 Da in the tea samples (on 35 d pile-fermentation) reached 2604, accounting for 58.47% of the total number of peptides identified in the tea samples at the same stage, while the number of peptides with a molecular weight of >800 Da was small, accounting for only 2.87% (35 d) to 5.23% (0 d) of the total number of small molecular peptides (Figure 4A). Similar results were also found in the identification of peptides in the ripening process of peach fruit, which may be related to the extraction of peptides, the method of instrument detection and the abundance of peptides with corresponding molecular weight [30]. The number of peptides identified in tea samples at different pile-fermentation stages was higher in hexapeptides, heptapeptides and octopeptides, with the number of hexapeptides > heptapeptides > octopeptides. Even the number of hexapeptides in the initial tea sample of the pile-fermentation was at least 1134, accounting for 52.50% of the total number of peptides identified in the tea samples at the same stage. The number of hexapeptides in tea samples increased sharply to 2257 (on 35 d of pile-fermentation), accounting for 50.68% of the total number of peptides in tea samples at this pile-fermentation stage, and then the number of hexapeptides began to decline, as shown in Figure 4B. The sharp increase in the number of hexapeptides was caused by the massive degradation of proteins and macromolecular peptides in tea under the action of protease, etc., and dynamic changes in the number of hexapeptides were highly positively correlated with the microbial populations and the activities of protease (unpublished data) produced by the relevant microbial populations during the process of pile-fermentation.

### 3.3. Principal Component Analysis and OPLS-DA of Small Molecular Peptides in Tea Samples from Different Pile-Fermentation Stages

**Figure 5:**
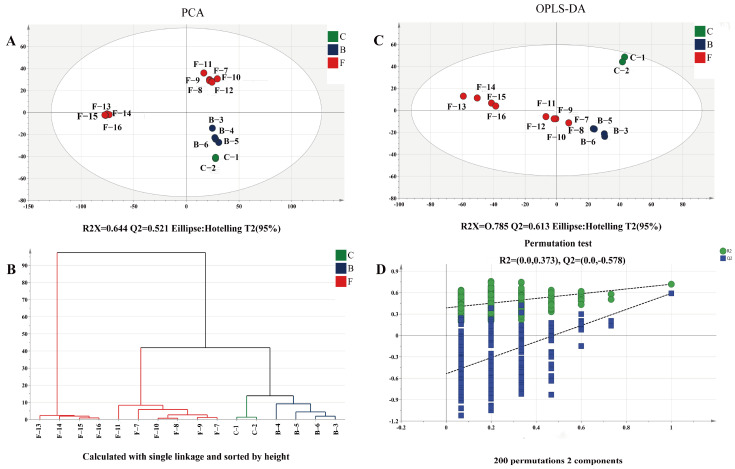
Analysis of peptide components in tea samples at different pile-fermentation stages of post-fermented tea (**A**), principal component analysis; (**B**), orthogonal partial least square-discriminate analysis, OPLS-DA; (**C**), cluster analysis; (**D**), permutation test).

The peptide components of tea samples were analyzed by nanoLC-MS/MS, and a total of 4563 peptides were successfully annotated in tea samples at different pile-fermentation stages of post-fermented tea according to the mass spectrum signal. The method of projection was used to reduce the dimensionality of complex data, and the distribution trend among samples could be visually displayed through principal component analysis [53]. Principal component analysis of small molecular peptides in tea samples at different pile-fermentation stages was carried out, the R^2^X = 0.644 and Q^2^ = 0.521 of the fitting curve were greater than 0.5, and all samples were within the 95% confidence interval. From the PCA score plot in Figure 5A, it can be seen that the samples in each group were relatively centralized, and the tea sample groups from eight different pile-fermentation stages showed a relatively obvious separation trend, which were separated into five parts in the vertical direction of the first principal component axis and the horizontal direction of the second principal component axis: C-1–C-2, B-3–B-4, B-5–B-6, F-7–F-12 and F-13–F-16, which indicated that there were similarities and differences in peptide composition among tea sample groups from different pile-fermentation stages.

Orthogonal partial least square discriminant analysis (OPLS-DA) was used to analyze the peptide composition of the tea sample groups at different pile-fermentation stages, and a more intuitive mathematical model to distinguish the difference in peptide composition between different groups could be obtained (Figure 5B). The R^2^X = 0.785 and Q^2^ = 0.613 of the fitting curve were greater than 0.5, indicating that the established model could better represent the original data and had good predictability. As also shown in Figure 5B, the tea sample groups of eight different pile-fermentation stages were divided into three parts: C-1–C-2, F-7–F-12, B-3–B-6 and F-13–F-16, respectively, representing the similarity and difference in peptide composition in tea samples at different pile-fermentation stages. To test the validity of the original model and the existence of an overfitting phenomenon, a replacement test (*n* = 200) was conducted on the data. The regression curve of Q^2^ intersected with the negative axis of the *Y*-axis, indicating that there was no overfitting phenomenon and the fitting of the established model was effective (shown in Figure 5D). According to the previous studies on the dynamic changes in microbial abundance (unpublished data), the pile-fermentation process was divided into three stages: the bacterial growth stage, that is, the early pile-fermentation stage (0–14 d); the middle stage of pile-fermentation (14–42 d), characterized by the rapid growth of filamentous fungi; and the late stage of pile-fermentation (42–49 d) with the active growth of fungi (represented by yeasts) and bacteria (represented by *bacillus* spp). The clustering results of tea samples at different pile-fermentation stages based on the difference in peptide composition by OPLS-DA (shown in Figure 5D) were consistent with the previous clustering results of tea samples based on microbial periodic differences by Luo et al. [9], further confirming that the difference in peptide composition during the pile-fermentation process may be caused by the dynamic growth of microbial populations.

### 3.4. Major Small Molecular Peptides and Their Biological Sources at Different Pile-Fermentation Stages

The label-free technique can be used to quantitatively analyze the dynamic changes in polypeptides [54]. In this study, orthogonal partial least squares discriminant analysis (OPLS-DA) was used to identify the peptide components that measured the difference between tea samples at different pile-fermentation stages. A total of 156 small molecular peptides with a relative abundance percentage > 0.1% were screened from peptides identified in tea samples at eight different pile-fermentation stages. By comparing with standards and searching various available proteome databases, 156 major peptides were found to be likely derived from 148 precursor proteins produced by 16 organisms at the genus level. In general, the biological sources of peptides could be divided into two categories: tea peptides (accounted for 49.36%) and microbial peptides. In total, 77 tea peptides were derived from the degradation of tea proteins, such as GRGDEEEL, ESIQPSD and GPLSGD, PPSYLTGE, PLYPGG, PSYLTGEF, GEFPGD had the common precursor protein, a cytoplasmic protein (A0A7J7I8L9) (for GRGDEEEL and ESIQPSD) and a protein in chloroplast membrane (A0A4S4E216) (for GPLSGD, PPSYLTGE, PLYPGG, PSYLTGEF and GEFPGD), respectively. On the one hand, the diversity of tea protein cleavage sites may lead to the production of multiple peptides [51], on the other hand, the specificity of hydrolase to the cleavage sites may be the main reason for the stability of peptides during the pile-fermentation process. Microbial peptides (79 peptides) were identified as likely derived from *Aspergillus*, *Penicillium*, *Yeast*, *Bacillus*, *Paenibacillus* and other genera via proteolytic processes, among which the number of peptides derived from the precursor protein produced by *Penicillium*, *Bacillus* and *Aspergillus* were relatively larger, and the number of peptides identified from *Penicillium* (15.38% of the total number of 156 major peptides) > *Bacillus* (11.54%) > *Aspergillus* (7.05%).

**Figure 6:**
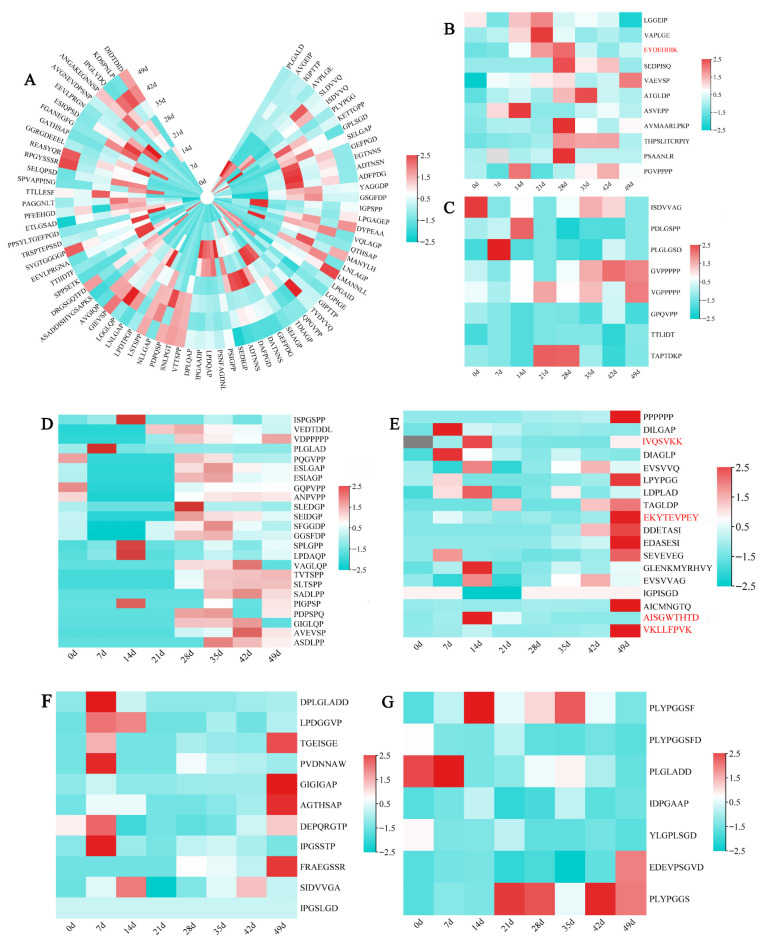
Heat map analysis of 156 major peptides (relative abundance percentage > 0.1%) from different biological sources at different pile-fermentation stages. Biological source of 156 major peptides: (**A**) *Camellia sinensis* var. sinensis; (**B**) *Aspergillus* sp.; (**C**) yeasts; (**D**) *Penicillium* sp.; (**E**) *Bacillus* sp.; (**F**) *Paenibacillus* sp.; (**G**) others. Red font refers to the peptide derived from the degradation of antimicrobial peptide.

According to the relative abundance percentage, a heat map analysis of major peptides derived from the same organisms (at level of genus) was performed as shown in Figure 6. Some peptides were determined to be present in the initial tea samples before pile-fermentation, indicating that these peptides were inherent in tea (Figure 6A). With the extension of pile-fermentation time, the abundance of major peptides from tea and *Aspergillus* sp. increased during the early and middle stages of pile-fermentation, and the abundance of major peptides from tea and *Aspergillus* sp. reached the maximum at 28 days of pile-fermentation and began to decline after 28 days of pile-fermentation (Figure 6B). The abundances of peptides from *Penicillium*, Yeast, *Bacillus* and *Paenibacillus* changed with almost the same trend, and they all had higher abundances at 7–14 d and 28–49 d of the pile-fermentation, which might be related to the rapid growth of these microorganisms adapted to the environmental conditions of the pile-fermentation (Figure 6C–G). The changes in the abundance of major peptides derived from tea plants and microorganisms reconfirmed the description of the three stages of microbial growth and reproduction in the pile-fermentation process, as consistent with Luo et al. [10,39]. The dominant bacteria such as *Bacillus* in the early pile-fermentation stage (0–14 d), the dominant filamentous fungi such as *Aspergillus* in the middle stage of pile-fermentation (14–42 d), and the active yeasts and bacteria in the late stage (42–49 d), respectively participated in the formation of peptides, which should be responsible for the change in the diversity of peptides during the pile-fermentation process of post-fermented tea. Peptide compositions were diverse and constantly changing during the whole pile-fermentation process of post-fermented tea. This study further revealed that the change in peptide diversity was almost consistent with the change trend of microbial population in tea samples [2,32]. Peptides were the products of pile-fermentation or fermentation under the direct participation of microorganisms, or the auxiliary action of enzymes produced by microorganisms [32]. The dynamic changes in the composition and abundance of major peptides in tea samples during pile-fermentation showed significant differences, which may be related to the microbial population and its metabolic activities during fermentation [54,55]. The systematic attention to the dynamic changes in the composition and abundance of the above characteristic peptides (especially those produced in the process of pile-fermentation) during the pile-fermentation of post-fermented tea may provide new ideas for the regulation of flavor quality and safety quality of post-fermented tea during the pile-fermentation process.

### 3.5. Identification of Antifungal Peptide Among Major Small Molecular Peptides

#### 3.5.1. Five Fragments of Antifungal Peptides Found in Tea Samples During the Pile-Fermentation Process

In order to identify the antifungal peptides produced by the dominant microorganisms during the pile-fermentation process, the 156 peptide sequences (with relative abundance percentage > 0.1%) identified from different pile-fermentation stages were entered into the antimicrobial peptide database (AMP database) for homology search and blasted with the known antimicrobial peptide sequences. Although no peptide sequences were screened for complete alignment with known antimicrobial peptides, five peptide sequences from 156 major peptides were found to be highly similar to local fragments of known antifungal peptides (shown in Table 1). Based on peptidomics and peptide homology analysis, it was found that the biological origin of EYDEHHIK was fungus of the *Aspergillus* (at genus), and its sequence similarity rate at the same sequence position of the antifungal peptide (AP01561) produced by *A. niger* was found to be 100% in the comparison of the AMP database. IVQSVKK, EKYTEVPEY, AISGWTHTD and VKLLFPVK, originated from *Bacillus*, had 100% sequence similarity at the same sequence position of the antifungal peptides (AP02242, AP02336, AP01963 and AP02243) respectively produced by *Bacillus subtilis*, *B. laterosporus* and *B. brevis* in the comparison of the AMP database. Therefore, EYDEHHIK, IVQSVKK, EKYTEVPEY, AISGWTHTD, and VKLLFPVK could be considered as peptide fragments that were retained in tea after degradation of antifungal peptides produced by *Aspergillus* and *Bacillus*, and were witnesses of antifungal peptides produced by microorganisms during the pile-fermentation of post-fermented tea.

**Figure 7:**
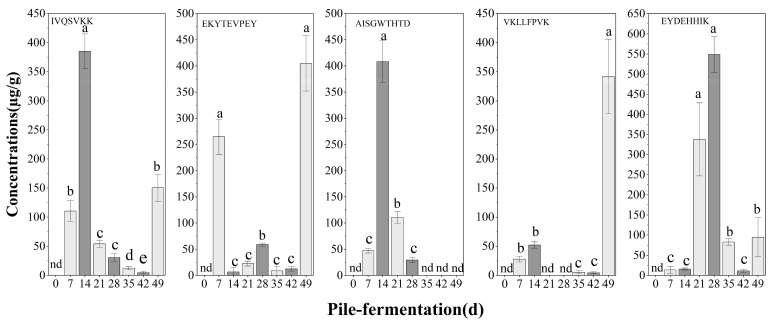
Relative concentration of antifungal peptide fragments during the pile-fermentation of post-fermented tea (nd: not detected in tea samples). The letters a, b, c, d and e on the bar chart are all significance markers for differences; different letters indicate significant differences between groups (*p* < 0.05), while the same letters indicate no significant differences between groups (*p* > 0.05).

The relative quantitative analysis of five antimicrobial peptide degradation fragments was performed by nanoLC-MS /MS using the synthetic peptide (VQLAGP) as the internal standard, and the dynamic changes in five antifungal peptide fragments during the pile-fermentation process are shown in Figure 7. None of the five fragments of antifungal peptides produced by *Aspergillus* and *Bacillus* were detected in tea raw material (i.e., at 0 d of pile-fermentation), which further indicated that they were produced by corresponding microbial metabolic activities during the pile-fermentation process of post-fermented tea. The fragments of antimicrobial peptides produced by *Bacillus* were mainly detected in the two stages: 7–21 d and 42–49 d of the pile-fermentation. The detected amount of IVQSVKK increased gradually from 7 to 14 days (the maximum value was 385.14 µg/g at 14 d), then decreased, and increased significantly again at 49 d of pile-fermentation; the detected amount of AISGWTHTD reached the maximum of 408.42 μg/g on the 14 d of the pile-fermentation; the detected amount of VKLLFPVK was small in the early stage of the pile-fermentation, and the detected amount of EKYTEVPEY was high on the 7 d of the pile-fermentation (264.61 μg/g at 7 d), and then decreased until the detected amount was again detected to reach the maximum 404.87 µg/g on the 49 d of the pile-fermentation; the detected amount of AISGWTHTD was 408.42 μg/g at 14 d; the detected amount of VKLLFPVK was small in the early stage and a maximum of 342.03 μg/g at 49 d, a high amount of EKYTEVPEY was detected at 7 d (up to 264.61 μg/g at 7 d), and then decreased on 49 d of the pile-fermentation; and EKYTEVPEY was detected again and reached the maximum value of 404.87 μg/g. The antimicrobial peptide fragment EYDEHHIK produced by *Aspergillus* was mainly detected from 21 d to 35 d of the pile-fermentation (with a maximum of 548.70 μg/g at 28 d).

Compared with the study of Luo et al. [9] on the dynamic changes in microbial population in the pile-fermentation process of post-fermented tea, it was found that the detection time of the five peptide fragments lagged the growth peak of *Bacillus* (0–7 d and 42–49 d) and *Aspergillus* (14–21 d) during the pile-fermentation of the post-fermented tea. For example, the growth peak of the *Bacillus* during the pile-fermentation was 0–7 d and 42–49 d, the four peptide fragments produced by *Bacillus* appeared on the 7–14 d and 49 d of the pile-fermentation, and the growth peak of *Aspergillus* and the detection time of the peptides produced by it also showed a similar rule. On one hand, the detection of the above peptide fragments suggested that the existence of antimicrobial peptides produced by *Bacillus* and *Aspergillus* in the process of the pile-fermentation played a role in inhibiting the growth of harmful fungi in the complex microbial environment of the pile-fermentation, on the other hand, the detection of the above peptide fragments also denoted the degradation of antimicrobial peptides: the greater the amount of peptide fragments detected, the greater the amount of antimicrobial peptides degraded and the lower the ability to inhibit harmful fungi. Therefore, the above peptide fragments can be used as an important index to measure their ability to inhibit harmful fungi in the pile-fermentation process of post-fermented tea.

#### 3.5.2. Potential Antifungal Activity of Other Small Molecular Peptides Extracted from Different Pile-Fermentation Stages

Interestingly, the inhibition results of tea peptide extracts on β-1,3-GS showed that some peptides inherent in tea might have certain antifungal activity (because the inhibition rate of peptides from the initial tea raw material of pile-fermentation on β-1,3-GS was 4.42%, as shown in Figure 1). In addition, there might be some other antifungal peptides of unknown origin yet to be revealed. Molecular docking was considered to be an effective and rapid method for preliminary identification of antifungal peptides by performing a computational docking analysis between peptides and β-1,3-GS [34]. In this study, molecular docking was used to screen the potential antifungal peptides in tea, and the further verification of the antifungal ability was conducted with the inhibition rate of synthetic peptides on β-1,3-GS, aiming to reveal the composition and source of antifungal peptides during the pile-fermentation process.

The fungus β-1,3-GS is a key enzyme in fungal cell wall biosynthesis, consisting of the catalytic subunit Fks1 and the essential regulatory factor, the small GTPase Rho1. Activated Rho1 (in its GTP-bound state) must bind to downstream effectors such as Fks1 to exert its function [56]. Peptides can block the effective interaction between Rho1 and Fks1 by occupying key binding sites on Rho1, thus inhibiting Fks1 activation and ultimately suppressing β-1,3-GS [57]. Although direct antifungal agents targeting Rho1 GTPase remain in the research stage, studies have demonstrated that Rho family GTPases play critical roles in fungal cell wall biosynthesis and morphogenesis. For instance, Rho4 in the maize smut fungus Ustilago maydis is essential for β-1,3-GS, cell wall integrity, and virulence [58]. Furthermore, Rho1 GTPase has been shown to be required for the regulation of the actin cytoskeleton in budding yeast. Antifungal strategies targeting the Rho1 pathway include inhibiting Rho1’s upstream GEF Rom2, targeting the Rho1 effector Pkc1, or enhancing Rho1-specific GAP activity to promote GTP hydrolysis [59].

**Figure 8:**
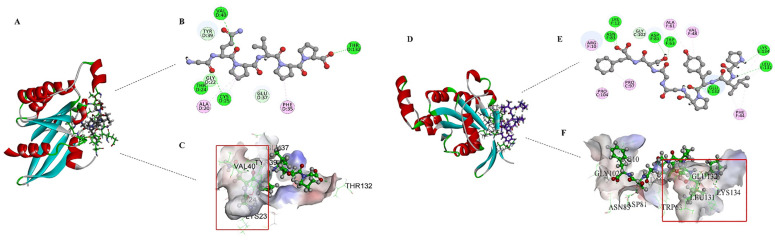
Molecular docking of GTPase protein with peptide ((**A**–**C**) GQPVPP; (**D**–**F**) PLYPGGSF). The binding pose (**A**,**D**) integration view, multicolor, (**B**,**E**) surface view, multicolor, and key interactions (**C**,**F**) of GQPVPP (**A**–**C**) and PLYPGGSF (**D**–**F**) with the active sites of a GTPase. The pink circles on residues indicate Van der Waals interactions; the green circles on residues indicate hydrogen bonds or ionic or polar interactions (**B**,**E**); the red rectangle represents the P-loop (**C**,**F**). The protein structure is displayed in a 3D format, with the purple stick model representing the ligand molecule and the green stick models representing the key amino acid residues within the protein’s active site that are involved in ligand binding.

A well-characterized yeast GTPase crystal structure (PDB ID: 3A58; 209 amino acids) was used to screen the antifungal peptide among the above 156 characteristic peptide sequences identified from different pile-fermentation stages (except for five peptides that had been identified as degradation products of known antifungal peptides) (Figure 8). Molecular simulation results showed that 27 out of 151 peptides could occupy the active site of GTPase, indicating that there was a small binding energy between peptides and GTPase. Binding energy is an index to evaluate the binding efficiency of ligands to biomacromolecules [34]. Binding energy < 0 indicates that the peptide has potential inhibition ability on GTPase. It is generally believed that the lower the binding energy, the stronger the inhibitory activity [60]. The binding energies of the 27 peptides identified in tea samples during the pile-fermentation process are shown in Table 2. The results showed that the binding energy of 27 peptides ranged from −0.54 to −9.01 kcal/mol, indicating that these peptides were effective inhibitors of GTPase. It might be thought that these peptides with lower binding energy played an important role in the inhibition of harmful fungi during the pile-fermentation process of post-fermented tea. These peptides with low binding energies were mainly likely derived from tea plants, which were the degradation products of tea peptides or tea proteins under the action of microbial enzymes, accounting for 51.85% of the total. The remainder were microbial peptides, which might be produced by such microorganisms as *Bacillus* sp. or formed by degradation of their extracellular proteins. For example, LAGGVAVIK, a peptide produced by *Bacillus subtilis*, which was previously reported by Zhang et al. to have antifungal activity, was also screened from the characteristic tea peptides by the method of molecular docking in this paper [34].

**Figure 9:**
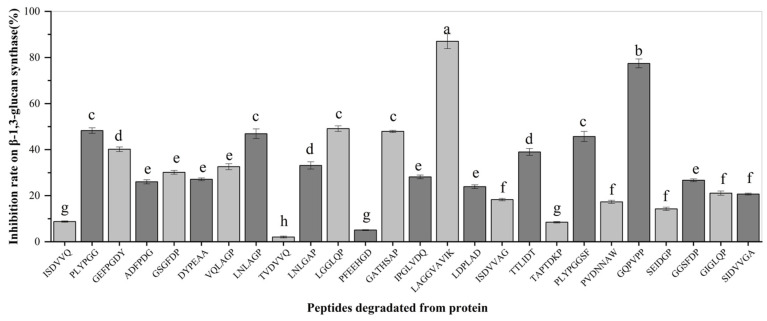
Inhibition rate of synthetic peptides on β-1,3-glucan synthase activity. The letters a, b, c, d, e, f, g and h on the bar chart are all significance markers for differences; different letters indicate significant differences between groups (*p* < 0.05), while the same letters in-dicate no significant differences between groups (*p* > 0.05).

A total of 27 peptides with binding energies < 0 kcal/mol identified by molecular docking were synthesized to validate their antifungal properties. β-1,3-GS extracted from the mycelium of *A. carbonarius* was used to evaluate the antifungal activity of synthetic peptides; the inhibitory effect of synthetic peptides with a concentration of 0.05 mg/mL on β-1,3-GS are shown in Figure 9. As stated in the Section 2, the uniform mass concentration was used for activity comparison, with molar concentration differences attributed to peptide molecular weight variations. Future studies will employ concentrations closer to physiological conditions and utilize purified peptide standards to systematically investigate the dose–response relationships and underlying antifungal mechanisms. The results showed that all synthetic peptides with binding energy < 0 kcal/mol (concentration of 0.05 mg/mL) had a certain inhibitory effect on β-1,3-GS, and the inhibitory effect of synthetic peptides on β-1,3-GS was negatively correlated with the binding energy of the selected peptide. The smaller the binding energy of the selected peptide was, the greater the inhibitory effect on β-1,3-GS. The inhibition rate of LAGGVAVIK with binding energy −9.81 kcal/mol on β-1,3-GS reached the maximum 87.04%, and the inhibition rate of TVDVVQ with binding energy −0.54 kcal/mol on β-1,3-GS was still 2.05%, indicating that these peptides with binding energy < 0 kcal/mol played an inhibitory role in the synthesis of β-1,3-glucan in cell wall construction during the growth of harmful fungi (such as *A. carbonarius*). These results further confirmed the antifungal activities of the selected peptides.

### 3.6. Correlation Among the Formation of Small Molecular Peptides Associated with Antifungal Activity, Dominant Microorganisms and Peptidase Activity During the Pile-Fermentation of Post-Fermented Tea

**Figure 10:**
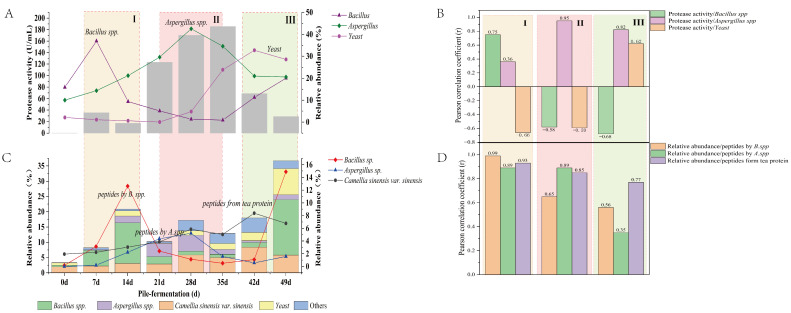
Relationship among dominant microorganisms, microbial peptidase activity and abundance of peptide with potential antifungal activity. (**A**) Relationship between microbial peptidases and the abundance of peptides with potential antifungal activity; (**B**) Correlation coefficient between microbial peptidases and the abundance of peptides with potential antifungal activity (|r| ≥ 0.2); (**C**) Relationship between dominant microorganisms and the abundance of peptides with potential antifungal activity; (**D**) Correlation coefficient between dominant microorganisms and the abundance of peptides with potential antifungal activity (|r| ≥ 0.2).

The small molecular peptides related to antifungal activity during the pile-fermentation of post-fermented tea could be divided into two categories. One was the enzymatic hydrolysis product of antifungal peptides produced by some microorganisms during the pile-fermentation process, which was the testimony of antifungal peptides produced by microorganisms. In this study, it was found that the microorganisms producing antifungal peptides mainly belong to the genera of *Bacillus* and *Aspergillus*, and the abundance of these small molecular peptides might be negatively correlated with the antibacterial activity of antifungal peptides produced by *Bacillus* and *Aspergillus*. The other was the small molecular peptides with potential antifungal activity formed by enzymatic hydrolysis of tea proteins or larger molecular peptides, and the content of which was positively correlated with antifungal activity. According to the dynamic analysis of the number of dominant microbial populations, microbial peptidase activity and small-molecule peptide abundance during the pile-fermentation process, it was found that there was a significant correlation among the number of dominant microbial populations, microbial peptidase activity and abundance of small-molecule peptides (as shown in Figure 10), and the pile-fermentation process was divided into three stages (I, II and III) according to the abundance of small molecular peptides and their sources. Stage I (from 7 days to 14 days of pile-fermentation): After the rapid growth of dominant bacteria represented by *Bacillus* on the tea substrate, a variety of small molecular peptides related to antifungal activity were found, and the maximum abundance of small molecular peptides appeared behind the peak of rapid growth of *Bacillus*. Small molecular peptides were degradation products of antifungal peptides produced by the strains of *Bacillus*, and their production was significantly positively correlated with peptidase activity. Peptidase is an enzyme that can break the peptide chains to hydrolyze proteins or macromolecular peptides. Peptidases can be divided into endopeptidases and exopeptidases according to the amino acids released during hydrolysis and the properties of the hydrolysis site. Microorganisms have been shown to be rich sources of peptidases [61]. The peptidases produced by *Bacillus* are mainly alkaline proteases [62], which may play an important role in the enzymatic hydrolysis of antimicrobial peptides produced by Bacillus to form related small molecular peptides in stage I of pile-fermentation. Stage II (from 21 days to 35 days of pile-fermentation): The dominant fungi, represented by *Aspergillus*, grew rapidly in the middle period of pile-fermentation. The small molecular peptides associated with antifungal activity found during this period were mainly derived from the degradation products of antifungal peptides produced by the strains of *Aspergillus* and the small molecular peptides formed by enzymatic hydrolysis of tea proteins or macromolecular peptides, and their formation showed a significant positive correlation with peptidase activity. Studies have shown that *Aspergillus*, *Penicillium* and *Bacillus* could produce peptidase [61]. They may play an important role in the formation of small molecular peptides at stage II. Stage III (from 42 days to 49 days of pile-fermentation): The dominant bacteria represented by *Bacillus* and the dominant fungi represented by *Yeasts* grew rapidly in the later period of pile-fermentation. The small molecular peptides related to antifungal activity came from two aspects: First, the antimicrobial peptides produced by *Bacillus* were degraded to form small molecular peptides. The other was that tea protein or macromolecular peptides were hydrolyzed by peptidase produced by microorganisms such as *Bacillus* and *Yeast* to form small molecular peptides with potential antifungal activity. Therefore, it could be inferred that the peptides that play an inhibitory role on harmful fungi at this stage mainly included antifungal peptides produced by *Bacillus* and small molecular peptides formed by the degradation of tea protein.

The above results on β-1,3-GS inhibition not only explain the antifungal mechanism of small molecular peptides in the pile-fermentation of Qingzhuan brick tea, but also have important reference significance for fungal control in other dark teas and even plant pathogenic fungus prevention and treatment. The pile-fermentation process of dark teas such as Pu’er ripe tea, Liubao tea and Fuzhuan tea shares similarities with that of the post-fermented tea in this study, all involving specific fungal communities [63]. The cell wall synthesis pathways of these fungi are highly conserved among different tea types, which means that the inhibition strategy targeting β-1,3-GS may also be effective for fungal control in these tea types [64]. Many plant pathogenic fungi, such as Fusarium graminearum, Botrytis cinerea and Pythium, rely heavily on intact cell wall structures for their pathogenicity [65]. Therefore, peptide or small-molecule inhibitors designed based on the β-1,3-GS target can specifically block the cell wall regeneration of these plant pathogenic fungi to achieve precise disease control [66]. For example, echinocandins targeting β-1,3-GS have been developed and applied as antifungal therapeutic drugs.

## 4. Conclusions

This study comprehensively elucidated the dynamic diversity, source characteristics, and antifungal activity mechanisms of small-molecule peptides during the pile-fermentation process of post-fermented tea. The dynamic variation pattern of small-molecule peptides during pile-fermentation was clarified: their content and variety exhibited a biphasic trend—initially increasing and subsequently decreasing—with peak abundance observed on day 35. Dominant peptide species were primarily hexapeptides and heptapeptides with molecular weights ranging from 600 to 800 Da, a distribution closely associated with microbial metabolic activities throughout pile-fermentation. The diverse origins of small-molecule peptides were revealed: 156 characteristic peptides originated from both microorganisms across 16 genera and endogenous tea plant proteins. Among the microbial contributions, *Penicillium*, *Bacillus* and *Aspergillus* exhibited the highest contributions, while tea plant-derived peptides accounted for 49.36% of the total. These findings demonstrate that the formation of the small-molecule peptidome is synergistically driven by microbial metabolism and the degradation of tea plant proteins. Two categories of antifungal-active small-molecule peptides were identified: one comprised degradation fragments of known antifungal peptides derived from *Aspergillus* and *Bacillus* (*n* = 5); the other consisted of novel antifungal peptides generated by the microbial enzymatic hydrolysis of tea proteins or macromolecular peptides (*n* = 27). The latter category was shown to bind to the active sites of β-1,3-GS through molecular docking, thereby effectively inhibiting enzyme function, with the inhibition rates reaching up to 87.04% for certain peptides. A mechanistic link between small-molecule peptide production and the fermentation environment was established: the biosynthesis and accumulation of antifungal-related peptides displayed distinct stage-specificity and were significantly positively correlated with the abundance of dominant microbial populations and peptidase activity. Furthermore, this study delineated differences in peptide production across the three stages of pile-fermentation, namely, the bacteria-dominated phase, the filamentous fungi-dominated phase, and the fungi–bacteria coexistence phase. This work not only expands the current understanding of antifungal active substances in post-fermented tea, but also reveals, for the first time, the natural inhibitory mechanism against harmful fungi during pile-fermentation from the perspective of peptidome dynamic evolution. It provides a solid theoretical foundation and technical support for enhancing the safety and quality of post-fermented tea through targeted fermentation process modulation, as well as for the development of novel natural antifungal peptide resources.

## Figures and Tables

**Table 1:** Peptide sequences probably derived from degradation fragments of known antifungal peptides.

Peptide Sequences in Tea Samples		Biological Sources	Antimicrobial Peptides (from AMP Database)		
			Original Sequence	Source	APD ID
Seq1	EYDEHHIK	*Aspergillus* spp.	LSKYGGECSVEHNTCTYLKGGKDHIVSCPSAANLRCKTERHHC**EYDHHIK**TVDCQTPV	*A. niger*	AP01561
Seq2	IVQSVKK	*Bacillus* spp.	TWATIGKT**IVQSVKK**CRTFTCGCSLGSCSNCN	*B. subtilis*	AP02242
Seq3	EKYTEVPEY		**EKYTEVPEYV**	*B. subtilis*	AP02336
Seq4	AISGWTHTD		ACQC**PDAISGWTHTD**YQCHGLENKMYRHVYAICMNGTQVYCRTEWGSSC	*B. laterosporus*	AP01963
Seq5	VKLLFPVK		**VKLLFPVK**LFP	*B. brevis*	AP02243

Note: Bold font indicates identity with the sequence.

**Table 2:** Peptides (from tea samples at different pile-fermentation stages) with binding energy < 0 identified by molecular docking with GTPase.

Polypeptide Sequence	FASTA Accession Number of the Master Protein	MW (Da)	Binding Energy (Kcal/mol)	Molar Concentration (μM)	Potential Organism	Polypeptide Sequence	FASTA Accession Number of the Master Protein	MW (Da)	Binding Energy (Kcal/mol)	Molar Concentration (μM)	Potential Organism
ISDVVQ	A0A7J7G177	660.35	−0.8	75.75	*Camellia* *Sinensis* var. *sinensis*	IPGLVDQ	A0A7J7HYZ9	741.41	−2.43	67.43	*Camellia Sinensis* var. *sinensis*
PLYPGG	A0A7J7GZ57	603.31	−5.91	82.91		LAGGVAIK	P28598	827.53	−9.81	60.42	*Bacillus* sp.
GEFPGD	A0A7J7GZ57	621.25	−4.75	79.96		LDPLAD	P46322	643.32	−0.98	77.72	
ADFPDG	A0A7J7HAH8	621.25	−2.13	80.48		ISDVVAG	A0AIH8VPP9	660.35	−1.79	75.71	*Yeasts*
GSGFDP	A0A7J7HLL3	579.24	−3.83	86.23		TTLIDT	Q05473	663.35	−3.52	75.37	
DYPEAA	A0A7J719S3	665.27	−2.83	75.15		TAPTDKP	A0A3M2CK58	729.37	−0.61	68.55	
VQLAGP	A0A7J7HZX4	584.34	−4.52	85.56		PLYPGGSF	A0A0B2EWG8	837.41	−4.05	59.71	*Paenibacillus* sp.
LNLAGP	A0A7J7GP28	584.34	−5.43	85.56		PVDNNAW	A0A932X597	815.36	−1.97	61.32	Others
TVDVVQ	A0A4S4EIB7	660.35	−0.54	75.71		GQPVPP	A0A8J8WIM5	594.32	−7.89	86.31	
LNLGAP	A0A4S4D6D3	584.34	−3.37	85.56		SEIDGP	A0A8J8WMJ0	617.27	−1.18	81.66	
LGGLQP	A0A7J7GP28	584.34	−4.96	85.56		GGSFDP	A0A8J8VWZ2	597.24	−3.99	84.12	
PFEEHGD	A0A067YP14	830.33	−0.62	60.21		GIGLQP	A0A8J8WB96	584.34	−2.39	85.56	
GATHSAP	A0A4S4DGS9	640.3	−4.27	78.08		SIDVVGA	A0A103BZA0	660.35	−2.79	75.71	

## Data Availability

The original contributions presented in this study are included in the article. Further inquiries can be directed to the corresponding authors.
